# Precision Aliphatic Polyesters via Segmer Assembly Polymerization

**DOI:** 10.3390/molecules23020452

**Published:** 2018-02-18

**Authors:** Fu-Rong Zeng, Yang Liang, Zi-Long Li

**Affiliations:** Department of Chemistry, College of Science, Huazhong Agricultural University, Wuhan 430070, Hubei, China; xiamoshang@webmail.hzau.edu.cn (F.-R.Z.); lysunshine@webmail.hzau.edu.cn (Y.L.)

**Keywords:** biodegradable polymer, precision aliphatic polyester, segmer assembly polymerization, periodic copolymer, microstructural control, sequence regulation, structure-property relation

## Abstract

Precise structure-property relation of a biodegradable polymer (e.g., aliphatic polyester) is anticipated only if monomer units and chiral centers are arranged in a defined primary sequence as a biomacromolecule. An emerging synthetic methodology, namely segmer assembly polymerization (SAP), is introduced in this paper to reveal the latest progress in polyester synthesis. Almost any periodic polyester envisioned can be synthesized via SAP using a programed linear or cyclic monomer. In this context, the macroscopic properties of a biodegradable polymer are fundamentally determined by microstructural information through a bottom-up approach. It can be highlighted that SAP ideally combines the precision of organic synthesis and the high efficiency of a polymerization reaction. Previously reported strategies including nucleophilic displacement, polyesterification, cross-metathesis polymerization (CMP), ring-opening polymerization (ROP), ring-opening metathesis polymerization (ROMP) and entropy-driven ring-opening metathesis polymerization (ED-ROMP) are critically reviewed in this paper to shed light on precision synthesis of aliphatic polyesters via SAP. Emerging yet challenging, SAP is a paradigm which reflects the convergence of organic and polymer chemistries and is also an efficient pathway to microstructural control. The current status, future challenges and promising trends in this realm are analyzed and discussed in this overview of the state-of-the-art.

## 1. Introduction

**Scheme 1:**
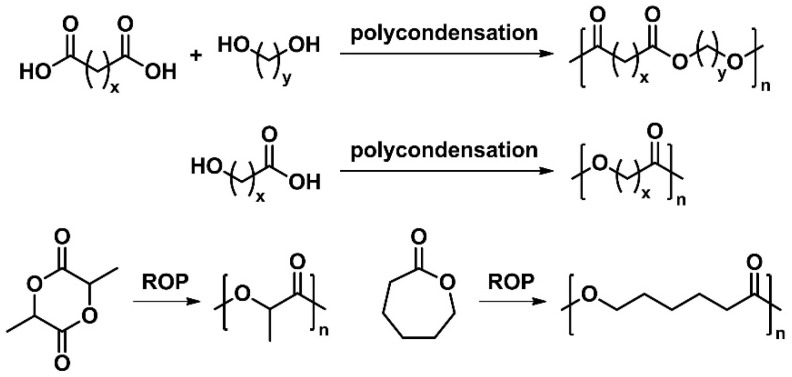
Polycondensation of diacids and diols (top) and *ω*-hydroxy acid (middle) as well as ROP of lactide and ε-caprolactone (bottom), respectively.

With the increasingly urgent demand for biocompatible and bioassimilable materials, synthetic biodegradable polymers elicit extensive attention from both academic and industrial communities [1]. In particular, as typical healthcare materials, aliphatic polyesters are widely used in the biomedical field [2]. Besides, aliphatic polyesters are among the most promising candidates for the replacement of petroleum-based polymers due to the wide availability of biorelevant monomers, ease of synthesis and biocompatibility of polymers and degradation products. Polycondensation and ring-opening polymerization (ROP) are the most widely used techniques for the synthesis of aliphatic polyesters (see Scheme 1). The easily operative polycondensation is widely used in industrial production with a broad range of starting materials [3]. Nevertheless, the generation of high molecular weight polymers is still an enormous challenge, and removal of small molecule byproducts such as water and alcohol is energy-intensive. Furthermore, due to side reactions including transesterification and degradation at high temperature and moderate functional group tolerance, construction of hierarchical topology and fabrication of functional materials are relatively difficult. On the other hand, ROP can be readily performed using organocatalysts, metal alkoxides or metal complexes as initiators [4,5,6,7,8,9,10,11,12,13,14]. Some ROP systems show living character [15,16], allowing for easy control of molecular weights and terminus structures of polymers. Nevertheless, ROP monomers for polyester synthesis are generally limited to lactones, lactides and recently emerging *O*-carboxyanhydrides [17]. Application areas of aliphatic polyesters range from clothing and packaging to agriculture and biomedicine [18,19,20,21,22,23,24,25,26,27,28]. For instance, aliphatic polyester-derived drug delivery vehicles, surgical sutures, implantable materials and tissue scaffolds have occupied more and more of the market share.

As remarkable bioengineering materials, poly(glycolic acid) (PGA), poly(lactic acid) (PLA) and poly(lactic-*co*-glycolic acid) (PLGA) attract tremendous research enthusiasm. These biomaterials are available from renewable resources, degradable requiring no specific enzymes and nontoxic both as polymers and as hydrolyzed monomers [29,30,31,32,33,34]. Typically, PGA, PLA and PLGA are synthesized via ROP of glycolide and lactide [35,36,37,38,39,40,41]. Applications of these materials in drug release, gene therapy, regenerative medicine and implants have been extensively investigated. Indeed, PLGA has become the preferred option for the manufacture of absorbable medical devices, drug-eluting stents and drug carriers [42,43,44,45]. Due to the excellent compatibility with the human body [46,47,48,49] and nontoxicity of degradation products [50], PGA, PLA and PLGA were tested for clinical use about four decades ago [51,52,53]. Finally, these polymers have been approved for clinical use by the FDA [54,55,56]. At present, biomedical and pharmaceutical applications of PLGA have expanded from drug delivery vehicles [57,58] and surgical sutures [59,60] to stem cell scaffolds [61] and tissue engineering matrices [62]. On the other hand, poly(ε-caprolactone) (PCL) represents another type of biocompatible and environmentally benign biomaterial. This polymer is widely utilized as a flexible molding material, which exhibits lower glass transition temperature (*T*_g_) and melting temperature (*T*_m_) yet higher tensile strength and elasticity than PLGA [63,64].

Despite great success, these polymers still face big challenges. For example, PLA exhibits good mechanical strength yet poor elasticity, and it shows a very low degradation rate in vivo (a few years) and low drug permeability [65,66]. On the contrary, PCL displays not only high elasticity and crystallinity but also remarkable drug permeability and slow degradation rates in vivo (several years) [27,67]. Thus, the material performances of PLA and PCL are partially complementary. On the other hand, decreased availability of drugs, diminished drug effect and burst release are reported if PLA- and PLGA-based drug carriers are applied in clinical use [68,69]. Indeed, these drug carriers display rapid degradation at the initial stage but slow degradation in the late period, which generally results in undesirable burst release of drugs along with incomplete clearance of remaining materials [70,71]. Fine tuning the degradation behaviors of aliphatic polyesters is definitely of great significance in this realm. However, these key profiles of aliphatic polyesters have been discovered to be influenced by various factors including, but not limited to, composition, topology, functionality, molar mass, microstructure (sequence and tacticity), morphology and water uptake [72,73,74,75]. Hence, it is not surprising to find that a precise structure–property relationship is still out of reach. Fortunately, previous strategies such as changing the feed ratio of monomer pairs, incorporation of new monomers and adjusting the ratio of racemic to stereopure lactic units are indeed significant to inspire upcoming work aimed at control of crystallinity and hydrophilicity of aliphatic polyesters [76,77,78,79,80,81]. Nevertheless, simultaneous control over sequence and tacticity has been the preferred option to establish a precise structure–property relationship, which in turn offers a great impetus to the development of precision aliphatic polyesters. For example, stereoselective aliphatic polyesters can be synthesized via ROP of epoxides and cyclic anhydrides by using discrete metal complexes [82,83]. At the same time, alternating polyesters were generated via ROP of enantiopure cyclic monomers by using syndioselective catalysts [84,85,86].

As for polymer chemistry, precise structure-property relation of polymer materials is anticipated only if monomer units and chiral centers are strictly arranged in a defined order on a polymer chain, and new synthetic methods are the prerequisites to achieve this goal. In order to fulfill sophisticated functions, polymers with complicated microstructures are urgently needed. It is worth noting that concepts borrowed from organic chemistry including chemo-, regio- and stereo-selectivity more and more frequently appear in polymer research publications. Indeed, the convergence of synthetic organic and polymer chemistries is a great impetus for the advancement of polymer synthesis [87]. Precision polymerization has become the mainstream of polymer science, and the ultimate goal is to realize control over sequence and tacticity using carefully designed physical and/or chemical means [88,89,90,91,92,93,94,95]. In this context, the macroscopic properties of polymeric materials are fundamentally determined by the microstructural information through a bottom-up approach.

The main objective of this review is to shed light on precision synthesis of aliphatic polyesters. Examples of sequence-controlled aliphatic polyesters via segmer assembly polymerization (SAP) are critically reviewed. The current status, future challenges and promising trends in this realm are analyzed and discussed in this overview of the state-of-the-art. The authors do not claim to give exhaustive coverage of the topic.

## 2. Segmer Assembly Polymerization (SAP)

**Scheme 2:**
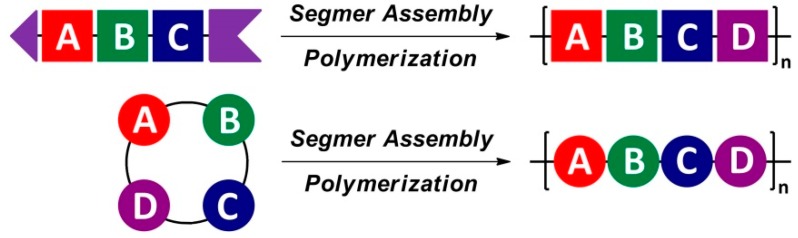
Schematic illustration of segmer assembly polymerization (SAP).

As stated above, macroscopic properties of polymer materials such as melting, crystallinity and degradation largely depend on sequence. Thanks to the rapid development of sequence-controlled polymerization during the last five years, sequence regulation and tacticity control come to reality by using conceptually new strategies. In particular, SAP paves the way towards easy and efficient synthesis of copolymers with periodic sequences [96]. As shown in Scheme 2, almost any periodic copolymers envisioned can be obtained starting from pre-organized linear or cyclic monomers with tailor-made microstructures. Encoded information such as sequence and tacticity within monomers is fully transferred to each repeating unit of periodic copolymer. Reactive terminuses are covalently bonded during SAP without affecting the encoded information, which is just the prerequisite of the aforementioned intact transference. New monomer units are generated via polymerization in some cases. Compared with chain-growth polymerization, SAP fundamentally overcomes the restriction of monomer structures, and polymerization is routinely conducted regardless of the reactivity of different monomers. On the other hand, in contrast to traditional step-growth polymerization, this universal strategy enables the synthesis of sophisticated sequence-controlled polymers. At present, it seems difficult to imagine sequence-regulated and stereo-specific polymers being obtained via direct copolymerization of relevant monomers in a batch mode, but organic chemistry offers an alternative to address the problem. The design, synthesis and purification of segmer bearing encoded sequences and tacticity can be conducted as organic chemists do, and applying the correct polymerization reaction is the only requirement to implement the strategy. SAP provides numerous possibilities for periodic copolymer synthesis, and serves as a paradigm to demonstrate the convergence of synthetic organic and polymer chemistries.

**Scheme 3:**
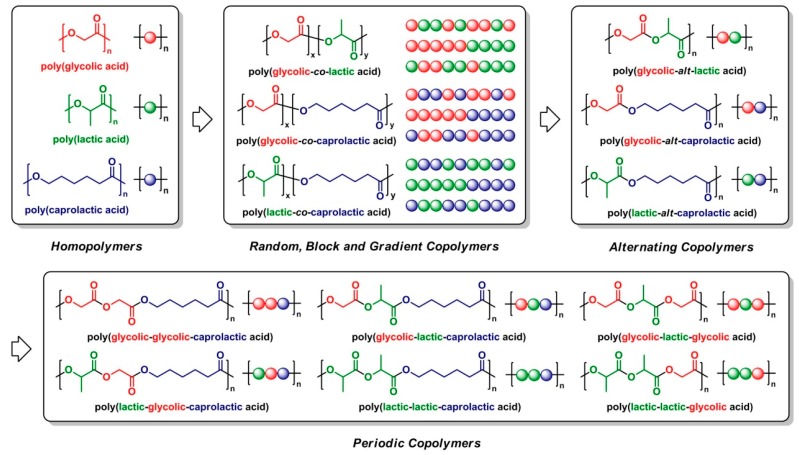
Evolution of aliphatic polyesters.

The arrangement of well-known monomer units such as glycolic, lactic and caprolactic acid on a polymer chain has become an increasingly appealing research field given that sequence regulation opens a new avenue to precise structure-property relationships and has become the preferred option for fine tuning material performance. As depicted in Scheme 3, the evolution of aliphatic polyesters goes from homopolymers to random, block, gradient, alternating and periodic copolymers. Most of the precision aliphatic polyesters have already been reported in the literature, and the ones with even more complex microstructures are anticipated to be obtainable in the near future. It is notable that the synthesis of alternating and periodic polyesters largely relies on SAP methodology. In this approach, starting from limited types of monomers, aliphatic polyesters will diversify into a sequence of products with different properties to meet the requirements of various circumstances.

Selected examples of precision synthesis of aliphatic polyesters via SAP are listed below, which can be categorized as step-growth polymerization (nucleophilic displacement, polyesterification and cross-metathesis polymerization (CMP)) together with chain-growth polymerization (ring-opening polymerization (ROP), ring-opening metathesis polymerization (ROMP) and entropy-driven ring-opening metathesis polymerization (ED-ROMP)).

## 3. Nucleophilic Displacement

**Scheme 4:**
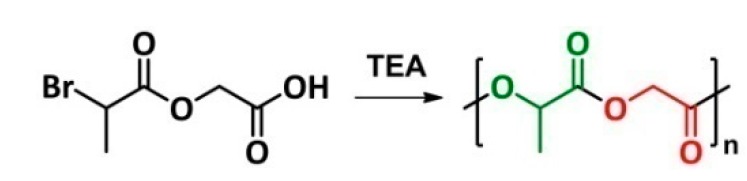
Synthesis of poly(lactic-*alt*-glycolic acid) via nucleophilic displacement.

Rebert [97] prepared poly(lactic-*alt*-glycolic acid) with *M*_n_ up to 66 kDa via polycondensation of *O*-(2′-bromopropionyl)glycolic acid as shown in Scheme 4. The preformed triethylammonium salt of the monomer was a nucleophile to displace bromine moieties to furnish polyester. This is an unusual polycondensation since hydrobromic acid is the byproduct instead of water. This method is readily applicable to the synthesis of diverse precision aliphatic polyesters.

## 4. Polyesterification

**Scheme 5:**
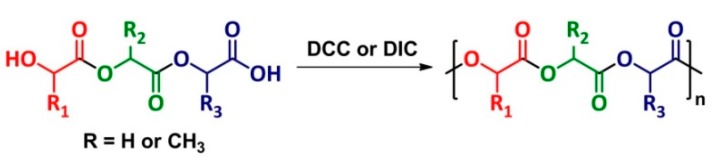
Precision synthesis of poly(lactic-*co*-glycolic acids) (PLGAs) via polyesterification.

Meyer and colleagues have recently developed a new method to furnish PLGAs of any targeted repeating sequence [98,99,100] and reported the sequence effects on NMR spectroscopy, solution-phase conformations and thermal properties of polymers [101]. As shown in Scheme 5, an AB-type monomer with designed sequence and tacticity is polymerized in the presence of a condensation reagent such as dicyclohexylcarbodiimide (DCC) and *N*,*N′*-Diisopropylcarbodiimide (DIC) in a stepwise manner to afford precision aliphatic polyester. This method is robust as evidenced by the high *M*_n_ values which are over 12 kDa in all cases. Likewise, this group proceeded to explore sequence effects by using polyesters comprised of glycolic, lactic and caprolactic acid units [102]. Meanwhile, they synthesized PLGA-type copolymers containing evenly distributed pendent groups [103]. It was found that sequenced PLGA showed a steady hydrolysis profile without abrupt changes in properties. In other words, sequenced PLGA degrades at a slower and more constant rate than its random counterpart [104]. This important observation is definitely helpful to establish a precise structure-property relationship and ultimately to decipher sequence-dependent degradation behavior. They have also systematically investigated the effects of sequences on degradation behaviors of PLGA microparticles and the encapsulation and release of rhodamine-B thereof [105]. The result reveals that a more gradual burst release is achieved by employing microparticles composed of sequenced PLGAs. Pioneered by this group, biomedical applications of microparticles and matrices composed of sequence-engineered PLGAs are currently under exploration [106,107]. Nevertheless, it is noteworthy that transesterification was occasionally observed during polymerization, thus disrupting microstructural periodicity. Furthermore, selective protection and deprotection are indispensable for monomer synthesis. On the other hand, crystalline alternating polyesters having lactic acid units were synthesized in a similar manner to that reported by Abe [108], and the copolymer melting temperature was closely related to the stereospecificity of the lactic acid unit.

## 5. Cross-Metathesis Polymerization (CMP)

**Scheme 6:**
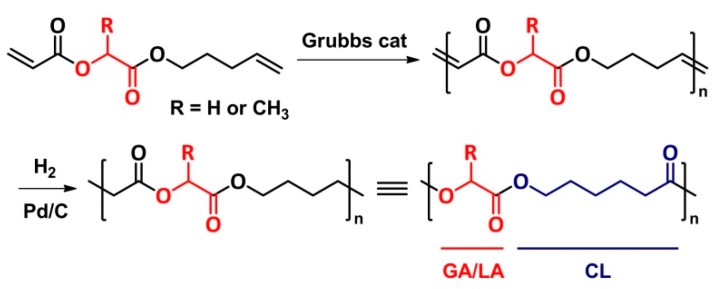
Precision aliphatic polyesters with alternating microstructures via cross-metathesis polymerization (CMP).

The incorporation of a polymerizable group, the key moiety for monomer design, is basically required for an SAP strategy where compatibility between the polymerization method employed and functional groups is a significant concern. In this regard, metathesis polymerization emerges as a promising candidate due to the ease of operation, high structural integrity and excellent tolerance to functional groups [109,110]. The authors [111] synthesized the first precision aliphatic polyesters with alternating microstructures by means of CMP. This polymerization technique is based on the high cross-metathesis tendency between acrylate and *α*-olefin motifs [112]. Due to its inherently alternating nature, CMP is particularly suitable for the synthesis of polyesters bearing β-substituted acrylate entities. Inspired by the traditional CMP of AB-type monomer bearing acrylate and α-olefin terminuses, as shown in Scheme 6, poly(glycolic-*alt*-caprolactic acid) and poly(lactic-*alt*-caprolactic acid) were generated simply by polymerizing pre-organized monomers that were synthesized in an efficient and straightforward fashion. It is noteworthy that the caprolactic acid unit was generated in situ via CMP followed by hydrogenation rather than incorporated into monomers. This carbon-carbon bond formation polymerization strategy fundamentally circumvents side reaction of transesterification and provides infinite possibility for rational design of periodic polyesters. Despite microstructural defects, this work opens a new avenue for sequence regulation and holds a promising future for the synthesis of precision aliphatic polyesters comprising well-known monomer units.

**Scheme 7:**
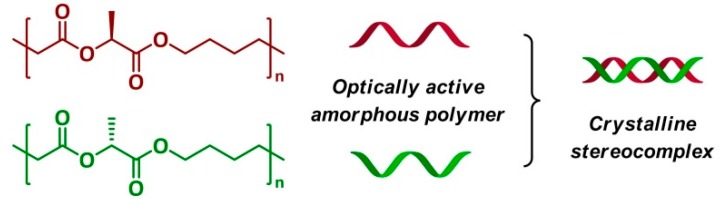
Optically active precision aliphatic polyesters via CMP.

To achieve simultaneous control over sequence and tacticity for this system, we [113] recently demonstrated the first example of optically active poly(lactic-*alt*-caprolactic acid) by means of CMP. As shown in Scheme 7, CMP of enantiopure monomers followed by hydrogenation readily afforded saturated polyesters composed of l- or d-lactic and caprolactic acid units. These two enantiomeric polyesters displayed opposite rotation values and mirror-imaged Cotton effects, corresponding to opposite helical conformations. Notably, stereocomplexity was generated simply by equimolar mixing of the two polyesters, which exhibited a sharp melting peak on a differential scanning calorimetry (DSC) curve at 45.7 °C. These results collectively indicate that the amorphous yet enantiomerically pure polyester becomes crystalline via stereocomplexation with its complementary enantiomer.

## 6. Ring-Opening Polymerization (ROP)

**Scheme 8:**
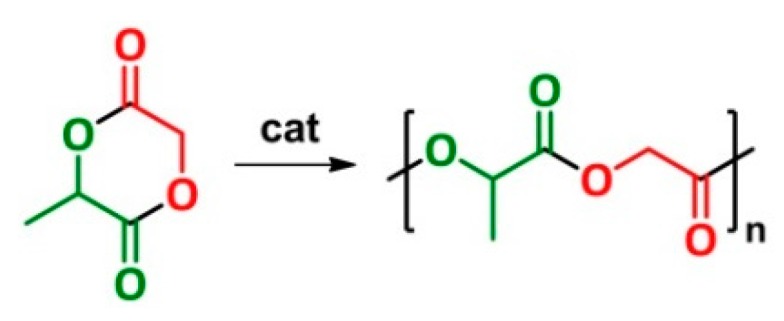
Synthesis of poly(lactic-*alt*-glycolic acid) via site-specific ring-opening polymerization (ROP).

As shown in Scheme 8, d,l-3-methylglycolide (MG) is a six-member lactone consisting of lactyl and glycolyl units. Early works on homopolymerization of MG using stannum-based catalysts failed to report detailed data of polymerization kinetics or verify the alternating microstructure of polymer [114,115]. Dong et al. [116] systematically investigated the preparation of poly(lactic-*alt*-glycolic acid) by using ROP of MG in the presence of stannous octoate. The ROP was conducted in bulk with a high reaction rate and high monomer conversion. The coordination-insertion mechanism and alternating sequence of polyesters were evidenced by NMR. Subsequently, they [117] reported living ROP of MG by using bimetallic (Al/Zn) μ-oxo alkoxide as an initiator. Notably, the cleavage of the acyl-oxygen bond of the monomer selectively takes place at the less hindered site, so that the obtained PLGA was primarily composed of alternating lactyl and glycolyl units. Moreover, this team [118] synthesized block and star-shaped copolymers using copolymerization of MG with ε-caprolactone and l-lactide, respectively. Afterwards, they [119] proceeded to investigate the in vitro degradation and controlled release behavior of precision PLGAs-derived microspheres. Although the alternating microstructure of the copolymer turned out to display no apparent effect on protein loading efficiency of microspheres, their original idea of sequence-controlled chain-growth polymerization by using a programmed cyclic monomer is indeed illuminating for future studies.

## 7. Ring-Opening Metathesis Polymerization (ROMP)

**Scheme 9:**
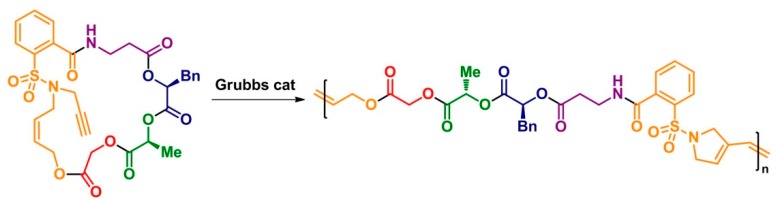
Ring-opening metathesis polymerization (ROMP) of sequenced macrocycle.

A general approach to sequence-controlled polymers via macrocyclic ring-opening metathesis polymerization (ROMP) was reported by Hawker [120]. As depicted in Scheme 9, this new method employs relay metathesis to promote ROMP of unstrained macrocycles. The success of this controlled and directional synthetic approach relies on the convergence of a small molecule “polymerization trigger” [121] and a diverse range of sequence-defined units. In particular, multiple α-hydroxy acid units are incorporated through ROMP of programmed macrocycles with a preformed sequence. This remarkable strategy combines the sequence precision of step-growth polymerization with the high tolerance to functionalities and livingness of ROMP. Nevertheless, the presence of “polymerization trigger” residue on polymer chains hinders direct biomedical use of this material.

## 8. Entropy-Driven Ring-Opening Metathesis Polymerization (ED-ROMP)

**Scheme 10:**
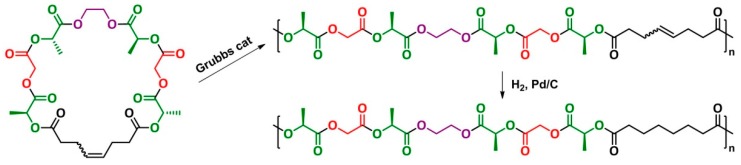
Sequence-controlled polyesters prepared via entropy-driven ring-opening metathesis polymerization (ED-ROMP).

Meyer et al. [122] employed ED-ROMP as a general approach to synthesize sequence-controlled copolymers bearing glycolic, lactic and caprolactic acid units. ED-ROMP is differentiated from ROMP in that it involves the ring-opening of low-strain or unstrained cyclic olefins to produce entropically favored polymers [123]. As depicted in Scheme 10, ED-ROMP of macrocycles containing an embedded sequence followed by hydrogenation readily generated saturated periodic polyesters. Notably, this strategy is categorized as chain-growth but not as living polymerization, and molecular weight can be easily adjusted by varying the monomer-to-catalyst ratio. High molecular weight polymers (*M*_n_ up to 60 kDa) were obtained with relatively low dispersities (*Ð* no more than 1.3). This approach should be applicable to periodic copolymer synthesis starting from any macrocycle bearing internal alkene moiety. Nevertheless, synthesis of macrocycles is still a big challenge where high dilution is basically required. Moreover, structurally asymmetric monomers are generally unsuitable for ED-ROMP due to the loss of sequence regularity.

## 9. Conclusions and Outlook

Synthetic methodologies for precision aliphatic polyesters are critically reviewed with an emphasis on SAP. This efficient and straightforward strategy makes it easy to synthesize almost any periodic polyester by using a sequence-defined and stereo-specific segmer. Nevertheless, one primary goal of this review is to call for further work from polymer scientists to push forward this emerging yet challenging research area. For example, precise structure-property relationships and sequence-dependent materials performance is still in its embryonic stage. Meanwhile, preorganization of segmers is a big challenge if selective protection/deprotection and tedious purification are inevitable. Therefore, the authors encourage our readers to read between the lines to foresee the future development of this field.

Considering the vast production and huge consumption of relevant commodities, it is inaccurate to say that aliphatic polyesters are still in their infancy. Actually, polyester synthesis has greatly evolved as a convenient, efficient and cost-effective protocol. Specifically, green chemistry plays an increasingly crucial role in polyester synthesis by virtue of the abundance of the relevant renewable resources [124] and wide application of green solvents. Meanwhile, synthesis of tailor-made polyesters can be conducted in a mild, selective and even atom-economic manner using new synthetic routes [125]. Furthermore, new chemical findings featuring high tolerance to polar groups renders straightforward the synthesis of functional polyesters [126]. Ultimately, synthesis of processable aliphatic polyesters with excellent thermal and mechanical properties represents a new challenge while preserving its biocompatible and eco-friendly nature. Great success has been achieved alongside the progress in synthesis. For example, more strategies for the synthesis of precision aliphatic polyesters are anticipated in the foreseeable future through SAP. On the other hand, chemical diversity is by no means the only option toward materials innovation. For instance, the approval of a new polymer for clinical use is rather laborious, so optimizing existing systems is seemingly wiser and more straightforward than developing new ones [127]. In this regard, Meyer’s contributions are good examples in that sequence regulation serves as an economic and pragmatic pathway towards conceptually new materials.

Synthetic polymers are still miles away from mimicking the complicated microstructures as well as the sophisticated functions of biopolymers. In contrast to organic synthesis of small molecules, the simultaneous control of chain length, sequence and tacticity is indeed a great challenge for polymer synthesis. For example, step-growth polymerization generally lacks control over molecular weight, and the generation of a more complex pattern is still out of reach for traditional ROP due to the at most dimeric form of relevant monomers. Notably, the emerging iterative approach for the synthesis of monodisperse and sequence-defined aliphatic polyester has become a vivid field of research [128]. These sequence-coded polyesters are highly designable. However, long chain products are generally unattainable within reasonable time frame and cost, and the overall yield becomes very low. Therefore, the synthesis of precision aliphatic polyesters with truly complicated microstructures still calls for conceptually new strategies. Hopefully, biological functions such as catalysis, substrate recognition and conformation transition will be expressed by precision aliphatic polyesters in the near future.
